# Ginsenoside-Rg1 Rescues Stress-Induced Depression-Like Behaviors via Suppression of Oxidative Stress and Neural Inflammation in Rats

**DOI:** 10.1155/2020/2325391

**Published:** 2020-03-18

**Authors:** Ye Li, Liyan Wang, Peng Wang, Cuiqin Fan, Ping Zhang, Jie Shen, Shu Yan Yu

**Affiliations:** ^1^Department of Physiology, Shandong University, School of Basic Medical Sciences, 44 Wenhuaxilu Road, Jinan, Shandong Province 250012, China; ^2^Morphological Experimental Center, Shandong University, School of Basic Medical Sciences, 44 Wenhuaxilu Road, Jinan, Shandong Province 250012, China; ^3^Department of Neurosurgery, Qilu Hospital of Shandong University, 107 Wenhuaxilu Road, Jinan, Shandong Province 250012, China; ^4^Shandong Provincial Key Laboratory of Mental Disorders, School of Basic Medical Sciences, 44 Wenhuaxilu Road, Jinan, Shandong Province 250012, China

## Abstract

Depression is an inflammatory-related condition, with the progression in neuronal damage resulting in major depression disorder. Ginsenoside-Rg1, a sterol extract from the herb *Panax ginseng*, has been shown to exert neuroprotective effects upon neurodegeneration disorders. However, whether ginsenoside-Rg1 confers antidepressant-like effects on neuroinflammation as associated with depression, as well as the possible mechanism involved in these neuroprotective effects, is currently unclear. In the present report, we show that treatment with ginsenoside-Rg1 (40 mg/kg, i.p.) significantly ameliorated depressive-like behaviors as induced by chronic unpredictable mild stress (CUMS) in a rat model of depression. Moreover, these CUMS rats treated with ginsenoside-Rg1 showed reductions in the levels of the oxidative stress products and the activity in the antioxidant stress kinase. Furthermore, CUMS rats treated with ginsenoside-Rg1 showed ameliorated neuroinflammation and associated neuronal apoptosis along with a reduction in dendritic spine atrophy and display of depressive behaviors. Taken together, the results of this study suggest that ginsenoside-Rg1 produces antidepressant-like effects in CUMS-exposed rats; and one of the mechanisms for these antidepressant-like effects of ginsenoside-Rg1 appears to involve protection against oxidative stress and thus the neuronal deterioration resulting from inflammatory responses. These findings provide evidence for the therapeutic potential of ginsenoside-Rg1 in the treatment of stress-related depression.

## 1. Introduction

Depression is considered a pervasive, worldwide neuropsychological disease associated with enormous medical and economic burdens on individuals and society [1]. Although significant progress has been made in the understanding and treatment of depression, approximately 30–50% of these patients remain unresponsive to available antidepressant treatments [2]. Moreover, most approved antidepressants used in clinical practice are accompanied with troublesome side effects [3]. This limited efficacy is likely attributable to the heterogeneity in biological mechanisms involved with depression. Therefore, identification of the underlying mechanisms of depression represents a critical area of investigation.

Recent evidence has indicated that depression is associated with increases in oxidative stress and inflammation, followed by a consequent activation in neuronal apoptosis. Such responses are, in part, due to environmental risk factors which then produce a neuroprogression of events resulting in the development of depression-like behaviors [4–6]. Results from clinical reports have shown that inflammation is closely associated with the pathogenesis of depression, as indicated by elevated levels of proinflammatory cytokines found in the peripheral circulation and some brain regions of depressed patients [7, 8]. Postmortem studies have revealed that major depression patients exhibit a reduced size in pyramidal neurons and a decreased number of GABAergic interneurons [9]. Within studies using animal models of depression, significant elevations in some critical proinflammatory cytokines including interleukin-1*β* (IL-1*β*), interferon-*γ* (IFN-*γ*), and tumor necrosis factor-*α* (TNF-*α*) were present, whereas treatments with some putative antidepressant drugs or neuroprotective reagents reduced the levels of these proinflammatory cytokines to varying degrees while also producing antidepressant-like effects [10, 11]. These results provide strong evidence indicating that inflammation-induced neuronal injury may represent a major risk factor for the development of depression. Therefore, targeting relevant neuronal mechanisms underlying this inflammation-induced pathogenesis of depression progression has the potential to serve as both a novel therapeutic and preventative approach in the treatment of this disorder.

Ginsenoside-Rg1 is a major active ingredient extracted from the herb *Panax ginseng*, that is, dammarane which is substituted by hydroxy groups at the 3beta, 6alpha, 12beta, and 20 <ital pro-S positions, in which the hydroxy groups at positions 6 and 20 have been converted to the corresponding beta-D-glucopyranosides and in which a double bond has been introduced at the 24-25 position. Ginsenoside-Rg1 has been shown to exert a neuroprotective function in Alzheimer's disease [12, 13] and also against cognitive and memory impairments in animal models of other neurological disorders [14, 15]. Unfortunately, the potential beneficial roles, and in particular the mechanisms, of ginsenoside-Rg1 in the pathogenesis of depression have not been reported in any detail. Results from previous studies within our laboratory have demonstrated that ginsenoside-Rg1 can exert antidepressant-like effects in rats subjected to chronic stress through its capacity to ameliorate synaptic deficits [16, 17]. However, the issue of whether ginsenoside-Rg1 could rescue neural injury via suppressing oxidative stress and thus the inflammatory or apoptotic responses associated with depressive behavior remains unknown. Therefore, in this report, we investigated some of the possible mechanisms through which ginsenoside-Rg1 protects against stress-induced neuronal injury within the hippocampal CA1 region in a rat model of depression.

The hippocampus, which recently has been identified as one of the key brain areas associated with depression and other psychiatric disorders, shows dendritic synaptic impairment and severe damage after chronic stress exposure [18, 19]. Chronic unpredictable mild stress (CUMS) serves as a means to generate an animal model which mimics the common stressors experienced in humans. Accordingly, this model has been widely used for investigating the pathophysiological mechanisms underlying depression including alterations in neuronal, endocrine, and behavioral responses [20].

Given this background information indicating the potential significance, but lack of data, on the protective effects of ginsenoside-Rg1 upon neuronal damage within the hippocampal CA1 region after CUMS, in this report, we directed our efforts at investigating the effects of this agent and, in specific, some of the mechanisms involved in its capacity to modulate the damage associated with depression.

## 2. Materials and Methods

### 2.1. Animals

All animal experimental procedures were approved by the Shandong University Animal Care and Use Committee and were performed according to the International Guiding Principles for Animal Research provided by the International Organizations of Medical Sciences Council. All efforts were made to minimize pain and numbers of animals used in these experiments. Male Wistar rats weighing 180-200 g were obtained from the Shandong University Animal Centre. Rats were housed in groups of four per cage under standard laboratory conditions with free access to food and water and maintained under a 12 h light/dark cycle (lights on 6:30 a.m., lights off 6:30 p.m.) for at least one week prior to experimental procedures. The study was not preregistered.

### 2.2. Rat Depression Model

The chronic unpredictable mild stress (CUMS) procedure was used to generate an animal model of depression as based on previously described procedures with minor modifications [21]. Briefly, rats were housed individually in a separate colony room and subjected to chronic stress over a 5-week period. The stress regime included physical restraint (2 h), cold swimming (5 min, 4°C), overnight illumination, cage shaking (2 h), 24 h food deprivation followed by 24 h water deprivation, and wet bedding (24 h). One of these stressors was applied daily to each rat in a random order for a 5-week period.

### 2.3. Drug Administration and Animal Groups

Ginsenoside-Rg1 was purchased from the National Institute for the Control of Pharmaceutical and Biological Products (Beijing, China) and was dissolved in sterile-endotoxin-free saline (NaCl, 0.9%) at a concentration of 20 mg/ml based on previous results [22]. Fluoxetine (Sigma, USA) was dissolved in 0.9% saline at a concentration of 20 mg/ml based on previous study [23]. Rats were randomly assigned to one of the following groups (*N* = 30/group): (1) control (nonstressed), (2) CUMS, (3) CUMS pretreated with ginsenoside-Rg1 (40 mg/kg; G-Rg1+CUMS), (4) CUMS pretreated with fluoxetine (40 mg/kg; FLX+CUMS), and (5) control treated with ginsenoside-Rg1 (control+G-Rg1). Ginsenoside-Rg1 or fluoxetine was administered daily via an intraperitoneal (i.p.) injection at 60 min prior to CUMS procedures over the 5-week CUMS period. The experimental schedule is presented in Supplementary [Supplementary-material supplementary-material-1].

### 2.4. Behavioral Tests

Behavioral tests were conducted on all animals after the 5 weeks of CUMS.

*Sucrose preference test.* The sucrose preference test was performed to assess anhedonia in these rats as described previously [21]. Briefly, rats were placed individually in cages with access to two bottles of 1% sucrose solution for the first 24 h, then one bottle was replaced with tap water for the second 24 h period. After this adaptation phase, rats were deprived of food and water for 24 h and then permitted free access to the two bottles, one containing 100 ml of sucrose solution (1%, *w*/*v*) and the other 100 ml of tap water for 3 h. The sucrose preference, which provides an index of anhedonia, was presented as follows: sucrose consumption/[water consumption + sucrose consumption] × 100%

*Forced swim test.* The forced swim test was performed to assess despair behavior in these rats as described previously [24, 25]. Briefly, in the training session, rats were placed individually in a cylinder (height: 80 cm, diameter: 30 cm, and temperature: 25°C) for 15 min of forced swimming. Twenty-four hours later, each rat was placed in the cylinder for a 5 min test session. The durations of immobility (floating except movements required to maintaining their head above the water), swimming, and struggling (climbing walls or diving) were scored by an observer blind as to the treatment group

### 2.5. Golgi Staining

One day after behavioral tests, six rats per group were used for the Golgi staining. The Golgi staining was performed to assess changes in neuronal dendrites and dendritic spines of CA1 neurons using the FD Rapid GolgiStain™ Kit (PK401, FD Neuro-Technologies, MD21041, USA) according to the manufacturers' instructions. Briefly, the rats were anaesthetized with sodium pentobarbital (150 mg/kg, i.p.), and their brains rapidly removed and immersed in the impregnation solution (*A*/*B* = 1 : 1, total 15 ml/rat) for two weeks. The brains were sectioned serially into 100 *μ*m coronal sections, cleaned in xylene, and cover-slipped with rhamsan gum for light microscopic observation. Apical dendrites of pyramidal neurons were chosen for morphological analysis. For each group, a minimum of 4 to 6 dendritic segments per neuron were randomly selected, and 5 pyramidal neurons were analyzed per rat. The number of spines was analyzed with use of the Image-Pro plus software.

### 2.6. Transmission Electron Microscopy (TEM) Analysis

Six rats from each group were anaesthetized, the DG (1 mm × 1 mm × 1 mm) was carefully dissected and placed in 2.5% glutaraldehyde at 4°C for 4 h. The tissue was then fixed with 1% osmium tetroxide for 1 h and subjected to a graded ethanol dehydration series. The tissue was then infiltrated with a mixture of one-half propylene oxide overnight and embedded in resin. Tissues were cut into ultrathin sections (70 nm thick), then stained with 4% uranyl acetate for 20 min and with 0.5% lead citrate for 5 min. Ultrastructures of the DG neurons were observed using transmission electron microscopy (Philips Tecnai 20 U-Twin, Holland). A minimum of 30 micrographs were randomly selected from each rat for analysis using the ImageJ analysis software (NIH, Scion Corporation, Frederick, MD).

### 2.7. Confocal Immunofluorescence Assay

Six rats from each group were anesthetized and perfused with 4% paraformaldehyde (PFA). The brains were dissected and postfixed in PFA overnight at 4°C followed by a graded dehydration. Brain samples were then cut into serial coronal frozen slices (30 *μ*m) by an oscillating blade microtome (Leica VT1200). Slices were incubated with the primary polyclonal anti-4-hidroxynonenal (4-HNE, 1 : 500, Abcam, Cambridge, UK), anti-8-OHdG (1 : 400, Abcam, Cambridge, UK), anti-active caspase-3 (1 : 300, Abcam, USA), anti-NeuN (1 : 400, Cell Signaling Technology, USA), anti-ionized calcium binding adaptor molecule-1 (Iba-1) (1 : 500, WAKO, Japan), and rabbit antiglial fibrillary acidic protein (GFAP) (1 : 100, ProteinTech, USA) followed by the Alexa Fluor488-conjugated goat anti-rabbit IgG secondary antibody or Alexa Fluor594-conjugated goat anti-mouse IgG secondary antibody (all 1 : 200, Sigma-Aldrich). For analysis of ROS production, slices were stained with 10 *μ*M dihydroethidium (DHE, Sigma, USA) at 37°C for 30 min. Mitochondrial ROS levels were measured with use of 10 *μ*M MitoSOX Red fluorescent dye for 15 min at room temperature in the dark. Hoechst 33258 (C0031) was purchased from Solarbio (Beijing, China) and stained for 5 min. Images were captured using a scanning laser confocal microscope (LSM780, Carl Zeiss, Germany). For fluorescence intensity quantification, 20x confocal images of fluorescence staining slides and the negative control slides of CA1 were acquired using identical settings and further analyzed using the Image-Pro Plus. The integrated optical density (IOD) of IHC signals was divided by area value of DAPI signals to derive the fluorescence signal intensity. Fluorescence intensities were expressed as a percent of control group. A minimum of six to eight images were selected from each rat for analysis.

### 2.8. Reverse Transcription PCR and Quantitative Real-Time PCR

Rats were decapitated 24 h after behavioral testing and DG regions were carefully dissected on ice. Total RNA was isolated using the RNA rapid extraction kit (Aidlab, China) according to the manufacturer's protocol. Total RNA was reverse transcribed into cDNA, using the Revert Aid First Strand cDNA Synthesis Kit, and was subsequently amplified by PCR with use of specific primers (Supplementary [Supplementary-material supplementary-material-1]). Reaction products were separated by electrophoresis and images were obtained using the Gel Image Analysis System (Bio-Rad, USA). Intensities of the bands were analyzed using Image-Pro Plus 6.0 software and values were normalized to GAPDH.

Quantitative real-time PCR was performed with use of the Bio-Rad IQ5 Real-Time PCR System (Bio-Rad, USA). The relative fold change in expression of miRNA was determined using the 2^−(*ΔΔ*Ct)^ method. GAPDH served as a loading control in each sample.

### 2.9. Western Blot Analysis

One day after behavioral testing, six rats per group were decapitated for western blot analysis. DG regions were carefully dissected and immediately homogenized in lysis buffer with a cocktail of protease inhibitors. Protein concentrations were measured using the BCA assay kit (Beyotime, China). Equal amounts of protein from each sample were electrophoretically separated on 12% SDS-PAGE gels and transferred to PVDF membranes for analysis. The primary antibodies used were polyclonal rabbit anti-CD45 (1 : 1000, Abcam, USA), anti-CD11b (1 : 1000, Abcam, USA), and anti-*β*-actin (1 : 8000, Santa Cruz Biotechnology). The secondary antibody was horseradish peroxidase-conjugated to mouse anti-rabbit/mouse IgG (1 : 5000, Santa Cruz Biotechnology). The highly sensitive enhanced chemiluminescence kit, ECL (GE Healthcare, Buckinghamshire, UK), was used for these determinations. Protein band densities were quantified using the ImageJ software (NIH, Scion Corporation, Frederick, MD) and were normalized to *β*-actin. Final data were expressed as a percent of controls.

### 2.10. Oxidative Stress Measurements

Activity of antioxidant enzymes within CA1 tissue was measured with the use of the superoxide dismutase (SOD) activity assay kit (No. A001-3) and glutathione peroxidase (GSH-Px) activity assay kit (No. A005). The measurement was performed using tissue homogenates with standard protocols. Briefly, the substrate stock solution with buffer was diluted at ratio of 1 : 200 and the enzyme stock solution with enzyme was diluted at ratio of 1 : 10, then mixed sufficiently to confirm complete contact between samples and reagents and incubated at 37°C for 20 min. Finally, the mixture was to measure absorbance at 450 nm using an enzyme-labeled instrument. The contents of MDA (No. A003-1) and NO (No. A013-2) were measured using the assay kits according to the manufacturers' guidelines. The MDA analysis was performed using tissue homogenates with standard protocols. Mixed reagent was placed in 95°C water bath for 40 min, cooled in tap water, and centrifuged at 3500~4000 rpm for 10 min. Then transfer supernatant in cuvettes of 1 cm light path to measure the absorbance of all tubes at 532 nm. For levels of NO measurement, nitrate was converted to nitrite with aspergillus nitrite reductase, and the total nitrite was measured with the Griess reagent. After a 10 min incubation period at room temperature, the absorbance was determined at 543 nm using a spectrophotometer. Six rats per group were used for the experiments. All assay kits were purchased from Jiancheng Inc. (Nanjing, China).

### 2.11. Statistics

All statistical procedures were performed with use of SPSS version 13.0. One-way ANOVA was used to establish differences among groups of animals treated with drugs alone and was followed by Tukey's test for post hoc analysis. Other data were analyzed statistically by two-way ANOVA for multiple comparisons with pretreatment (CUMS × drug), and the post hoc Bonferroni test was conducted to determine differences between specific groups. All data were expressed as the means ± SEM. Differences with a *P* < 0.05 were considered statistically significant.

## 3. Results

### 3.1. Ginsenoside-Rg1 Alleviates Depression-Like Behaviors in CUMS Rats

**Figure 1:**
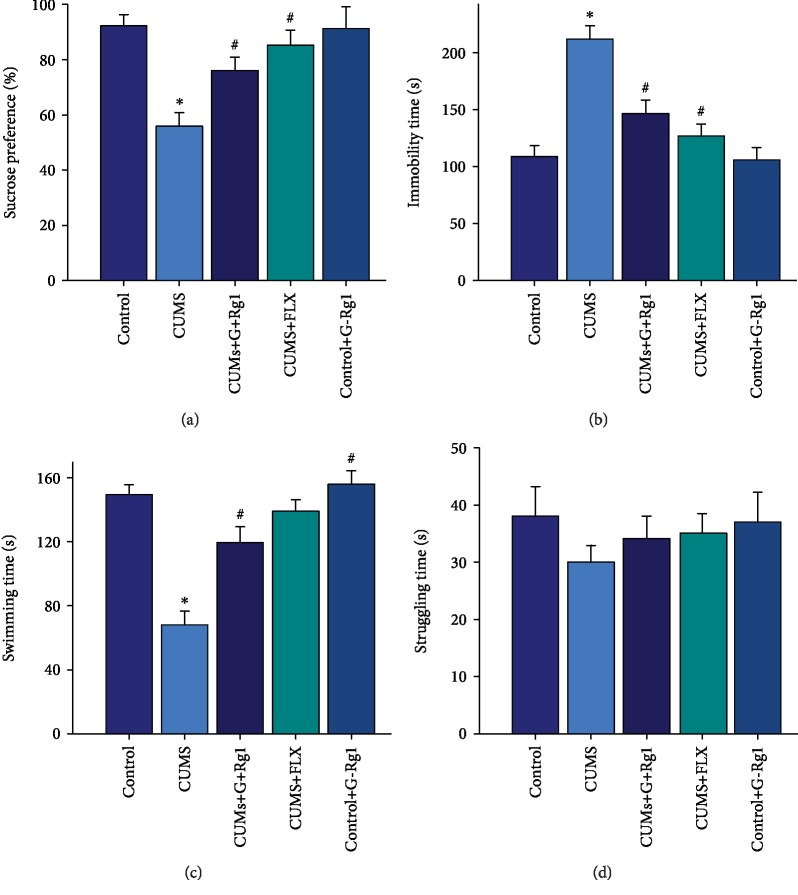
Ginsenoside-Rg1 ameliorates depression-like behaviors induced by CUMS exposure. (a) Pretreatment with ginsenoside-Rg1 (40 mg/kg) or fluoxetine (40 mg/kg) prevented the decreased consumption of sucrose solution in CUMS rats. (b) Pretreatment with ginsenoside-Rg1 or fluoxetine reversed the increases in immobility times of CUMS-exposed rats in the forced swim test. (c) Pretreatment with ginsenoside-Rg1 or fluoxetine reversed the decreases in swimming times of CUMS-exposed rats. (d) No statistically significant differences were obtained among the groups with regard to struggling times in the forced swim test. All values are presented as means ± SEM (*N* = 30). ^∗^*P* < 0.05, compared to the control group; ^#^*P* < 0.05, compared to the CUMS group. G-Rg1: ginsenoside-Rg1; FLX: fluoxetine; SPT: sucrose preference test; FST: forced swim test.

Results from the sucrose preference test showed that the percent of sucrose consumption was significantly different among the four groups (*F*_(3, 68)_ = 18.63, *P* < 0.05). Post hoc analysis revealed that the CUMS group showed a lower sucrose consumption percent as compared with that of the nonstressed control group (*P* < 0.05). Ginsenoside-Rg1 pretreatment significantly ameliorated anhedonia in CUMS rats (*P* < 0.05), in which effects were similar to that in response to treatment with the classic antidepressant, fluoxetine (*P* < 0.05; Figure 1(a)). With regard to the forced swim test, immobility times of CUMS rats were significantly increased (Figure 1(b)); that is, swimming times were significantly decreased (Figure 1(c)), as compared with the nonstressed control group (*P* < 0.05). Such responses denote behavioral despair, another core symptom of depression. However, ginsenoside-Rg1 treatment effectively alleviated this behavioral despair as indicated by decreased immobility and increased swimming durations in CUMS rats (*P* < 0.05, for both). There were no statistically significant differences among these groups with regard to struggling times (*F*_(3, 68)_ = 0.58, *P* > 0.05) (Figure 1(d)). There was no significant difference between the ginsenoside-Rg1-treated nonstressed control group and the nonstressed control group (*P* > 0.05). The findings of these behavioral assays demonstrate a potential antidepressant-like effect of ginsenoside-Rg1 in this CUMS model of depression.

### 3.2. Ginsenoside-Rg1 Attenuates Oxidative Stress in the Hippocampal CA1 Region of Depressed Rats

**Figure 2:**
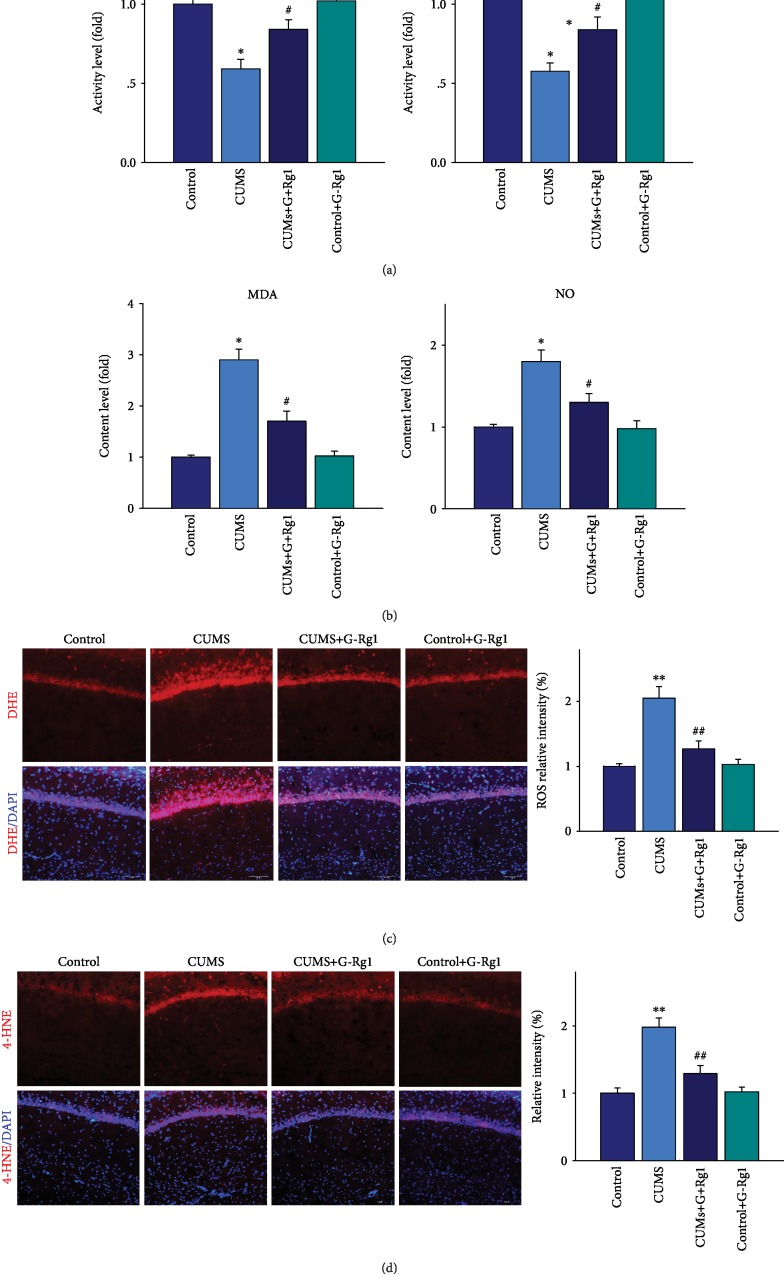
Ginsenoside-Rg1 suppresses oxidative stress in hippocampal CA1 regions of CUMS rats. (a) Pretreatment of ginsenoside-Rg1 elevates the decreased activity observed in the antioxidant enzymes, SOD, and GSH-pX, resulting from CUMS exposure. (b) Pretreatment of ginsenoside-Rg1 decreases MDA and NO contents in CUMS rats. (c) Ginsenoside-Rg1 alleviated the increased levels of DHE within the CA1 region caused by CUMS exposure. Nuclei (blue) are stained with DAPI. Scale bar is 50 *μ*m. (d) Ginsenoside-Rg1 alleviated the increased levels of 4-HNE within the CA1 region caused by CUMS exposure. Nuclei (blue) are stained with DAPI. Scale bar is 50 *μ*m. *N* = 6 per group. ^∗^*P* < 0.05 and ^∗∗^*P* < 0.01, compared to the control group; ^#^*P* < 0.05 and ^##^*P* < 0.01, compared to the CUMS group. G-Rg1: ginsenoside-Rg1.

To investigate the possible neuronal mechanisms of this antidepressant-like effect of ginsenoside-Rg1, we first examined changes in oxidative stress levels. We found that analysis of the overall activity of antioxidant enzymes within the hippocampal CA1 region was significantly different among the four groups (*F*_(3, 20)_ = 16.38, *P* < 0.05). A post hoc analysis revealed that the activity of antioxidant enzymes such as SOD and GSH-pX was significantly reduced in depressed rats as compared with that of the nonstressed control group (*P* < 0.05; Figure 2(a)); and levels of the oxidative stress products, malondialdehyde (MDA) and nitric oxide (NO), within the CA1 region were significantly increased after 5 weeks of CUMS exposure as compared with that of the nonstressed control group (*P* < 0.05; Figure 2(b)). In addition, DHE is specific for superoxide and hydrogen peroxide and was used as a fluorescent probe for the detection of ROS generation. Results showed that the ROS (*F*_(3, 20)_ = 17.62, *P* < 0.01) (Figure 2(c)) level within the CA1 region was significantly increased after 5 weeks of CUMS exposure as compared with that of the nonstressed control group. Meanwhile, 4-hydroxynonenal (4-HNE), an important marker of oxidative stress produced by lipid peroxidation in cells, was also significantly increased within the CA1 region of CUMS rats compared with the nonstressed control group (*F*_(3, 20)_ = 15.39, *P* < 0.01) (Figure 2(d)). In contrast, pretreatment of ginsenoside-Rg1 suppressed all of the above oxidative stress changes induced by CUMS exposure (*P* < 0.01). There was no significant difference between the ginsenoside-Rg1-treated nonstressed control group and the nonstressed control group (*P* > 0.05).

### 3.3. Ginsenoside-Rg1 Attenuates Mitochondria and DNA Oxidative Damage in the Hippocampal CA1 Region of Depressed Rats

**Figure 3:**
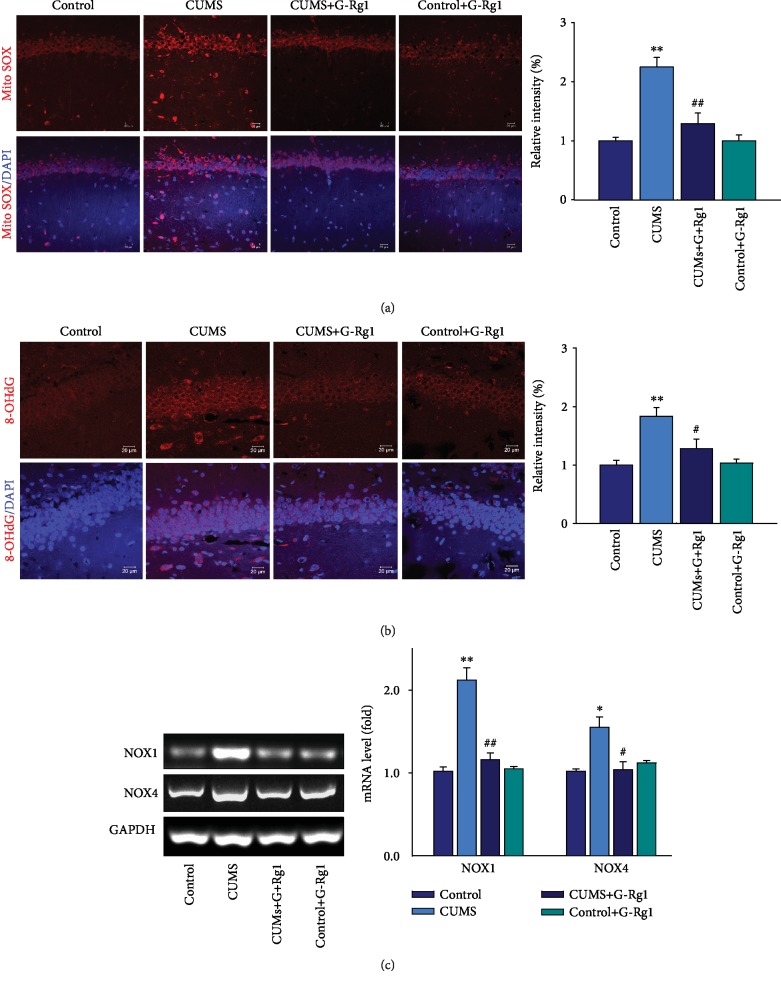
Ginsenoside-Rg1suppresses mitochondria and DNA oxidative damage in hippocampal CA1 regions of CUMS rats. (a) Ginsenoside-Rg1 alleviated the increased levels of MitoSOX. Nuclei (blue) are stained with DAPI. Scale bar is 20 *μ*m. (b) Ginsenoside-Rg1 alleviated the increased levels of 8-OHdG. Nuclei (blue) are stained with DAPI. Scale bar is 20 *μ*m. (c) RT-PCR analysis of NOX1 and NOX4 mRNA levels within each group. Band intensities were normalized to GAPDH. *N* = 6 per group. ^∗^*P* < 0.05 and ^∗∗^*P* < 0.01, compared to the control group; ^#^*P* < 0.05 and ^##^*P* < 0.01, compared to the CUMS group. G-Rg1: ginsenoside-Rg1.

Accumulation of oxidative stress products can result in mitochondrial and DNA damage, thus leading to cell death in both neurons and glia. Measures of MitoSOX provide an index of mitochondrial superoxide levels, and these were significantly increased within the CA1 region of depressed rats as compared with that in the nonstressed control group, while ginsenoside-Rg1 pretreatment effectively reversed these responses caused by CUMS exposure (*P* < 0.01; Figure 3(a)). Additionally, 8-oHdG expression, a marker of oxidative base damage, was significantly increased in depressed rats as compared with that in the nonstressed control group (*P* < 0.01), whereas decreased expression levels of 8-oHdG were observed in response to ginsenoside-Rg1 pretreatment (*P* < 0.05; Figure 3(b)). Such results imply that ginsenoside-Rg1 effectively reduced oxidative stress damage. Activated NADPH oxidase (NOX) represents the main pathway for superoxide production. Interestingly, we found that mRNA levels of NOX1 (*P* < 0.01) and NOX4 (*P* < 0.05) were significantly increased in depressed rats as compared with the nonstressed control group, an effect which was significantly attenuated by ginsenoside-Rg1 pretreatment (*P* < 0.05; Figure 3(c)).

In addition, there was no significant difference between the ginsenoside-Rg1-treated nonstressed control group and the nonstressed control group (*P* > 0.05). These results suggest that ginsenoside-Rg1 may protect against cellular oxidative damage via modulating the expression of some NOX family members.

### 3.4. Ginsenoside-Rg1 Decreases Inflammatory Response in the Hippocampal CA1 Region of Depressed Rats

**Figure 4:**
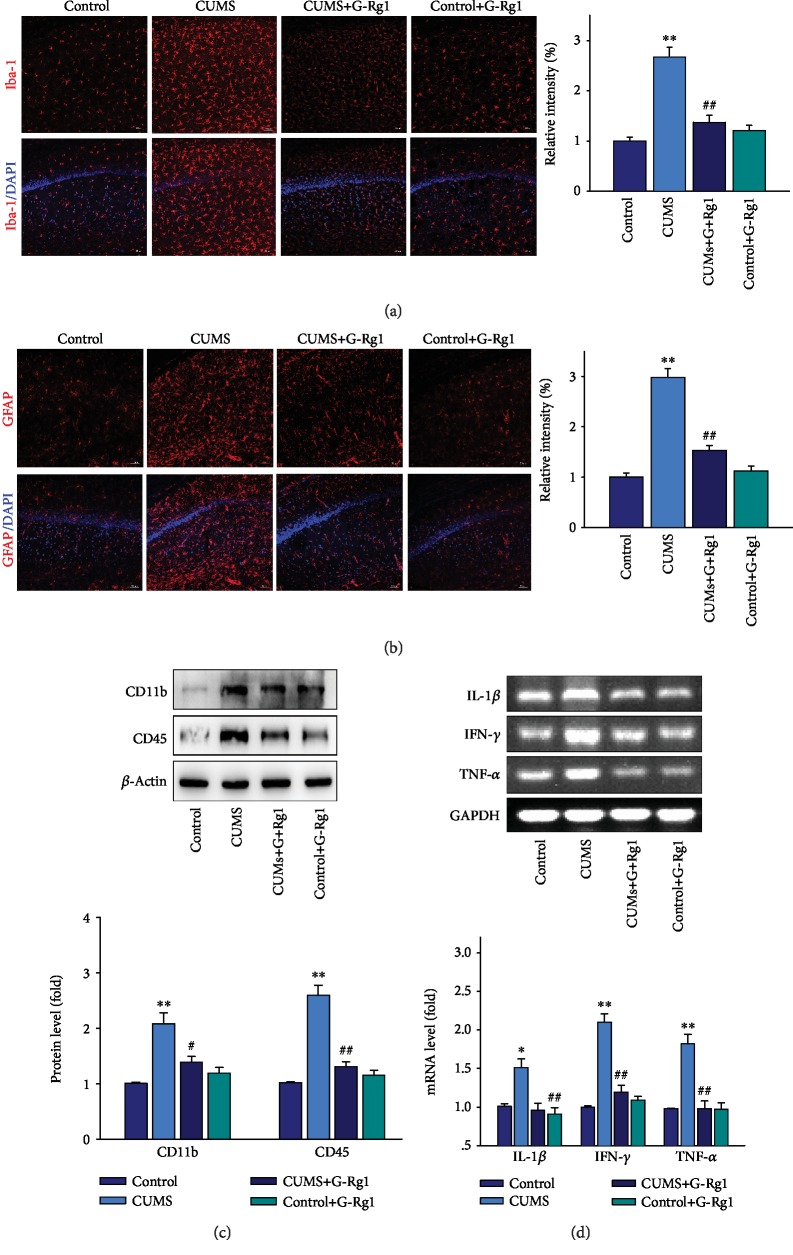
Ginsenoside-Rg1 suppresses glial activation and inflammatory cytokine expressions induced by CUMS exposure. (a) Immunofluorescence signals of Iba-1-positive microglial cells within the CA1 region. Scale bar is 50 *μ*m. (b) Immunofluorescence signals of GFAP-positive astrocytes within the CA1 region. Nuclei (blue) are stained with DAPI. Scale bar is 50 *μ*m. (c) Western blotting analysis of CD45 and CD11b protein expressions within each group. (d) RT-PCR assays of mRNA expression levels of IL-1*β*, TNF-*α*, and IFN-*γ* within each group. *N* = 6 per group. ^∗^*P* < 0.05 and ^∗∗^*P* < 0.01, compared to the control group; ^#^*P* < 0.05 and ^##^*P* < 0.01, compared to CUMS group. G-Rg1: ginsenoside-Rg1.

Neuroinflammation is considered a critical risk factor in the pathogenesis of depression. Here, we show that the number of Iba-1-positive microglia within the CA1 region was significantly different among the four groups (*F*_(3, 20)_ = 18.73, *P* < 0.01). The post hoc analysis revealed that 5 weeks of CUMS exposure significantly increased the number of activated microglia as compared with that of the nonstressed control group (*P* < 0.01; Figure 4(a)). Moreover, the number of GFAP-positive astroglia within the CA1 region was also increased in depressed rats as compared with the nonstressed control group (*P* < 0.01; Figure 4(b)). Results from the western blot analysis showed an overexpression of CD11b and CD45 proteins, two important microglial markers within the CA1 region of CUMS rats as compared with the nonstressed control group (*P* < 0.01 for both; Figure 4(c)). Consistently, mRNA expressions of several critical proinflammatory cytokines, such as IL-1*β*, IFN-*γ*, and TNF-*α*, within the CA1 region were all significantly increased as compared to that observed in the nonstressed control group (*P* < 0.01 for all; Figure 4(d)).

**Figure 5:**
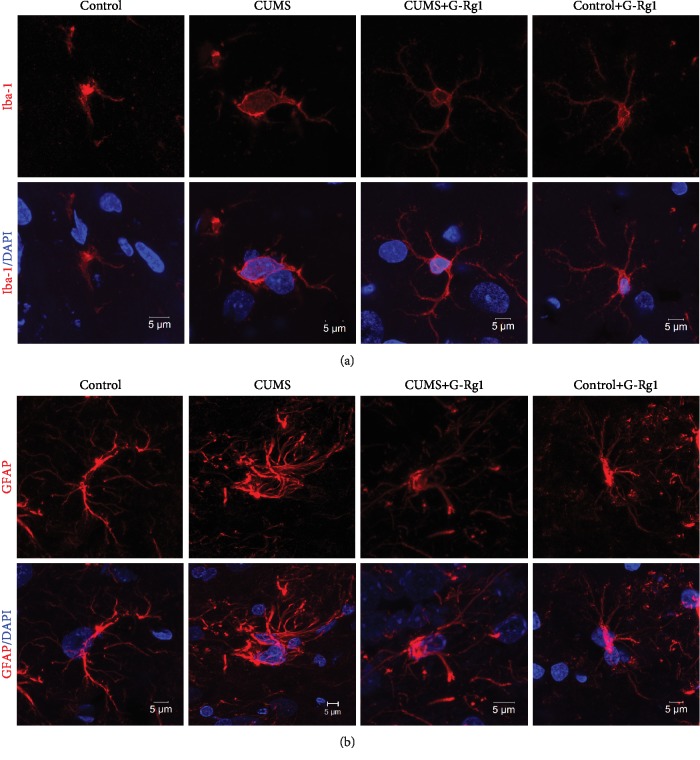
Ginsenoside-Rg1 alleviated morphological changes in activated glial caused by CUMS exposure. (a) Ginsenoside-Rg1 pretreatment alleviated morphological changes in activated microglia resulting from CUMS exposure. Scale bar is 5 *μ*m. (b) Ginsenoside-Rg1 pretreatment alleviated morphological changes in activated astrocytes resulting from CUMS exposure. Scale bar is 5 *μ*m.

Moreover, CUMS exposure produced changes in CA1 microglial morphology, with the neuroglia now showing substantial enlargements within the soma and ramified process retractions, alterations typical of activation as compared with that of the nonstressed control group (Figure 5(a)). Similar changes were also observed in reactive astrogliosis within the CA1 regions of CUMS rats as compared with the nonstressed control group (Figure 5(b)). Interestingly, as shown in Figures 4 and 5, ginsenoside-Rg1 pretreatment significantly suppressed these CUMS-induced glial activation, as well as producing a reduction in the expression of proinflammatory cytokines (*P* < 0.01). There was no significant difference between the ginsenoside-Rg1-treated nonstressed control group and the nonstressed control group (*P* > 0.05). These results indicate that ginsenoside-Rg1 produces a reduction in the increases of inflammatory responses that may contribute to depression.

### 3.5. Ginsenoside-Rg1 Decreases Apoptosis in the Hippocampal CA1 Region of Depressed Rats

**Figure 6:**
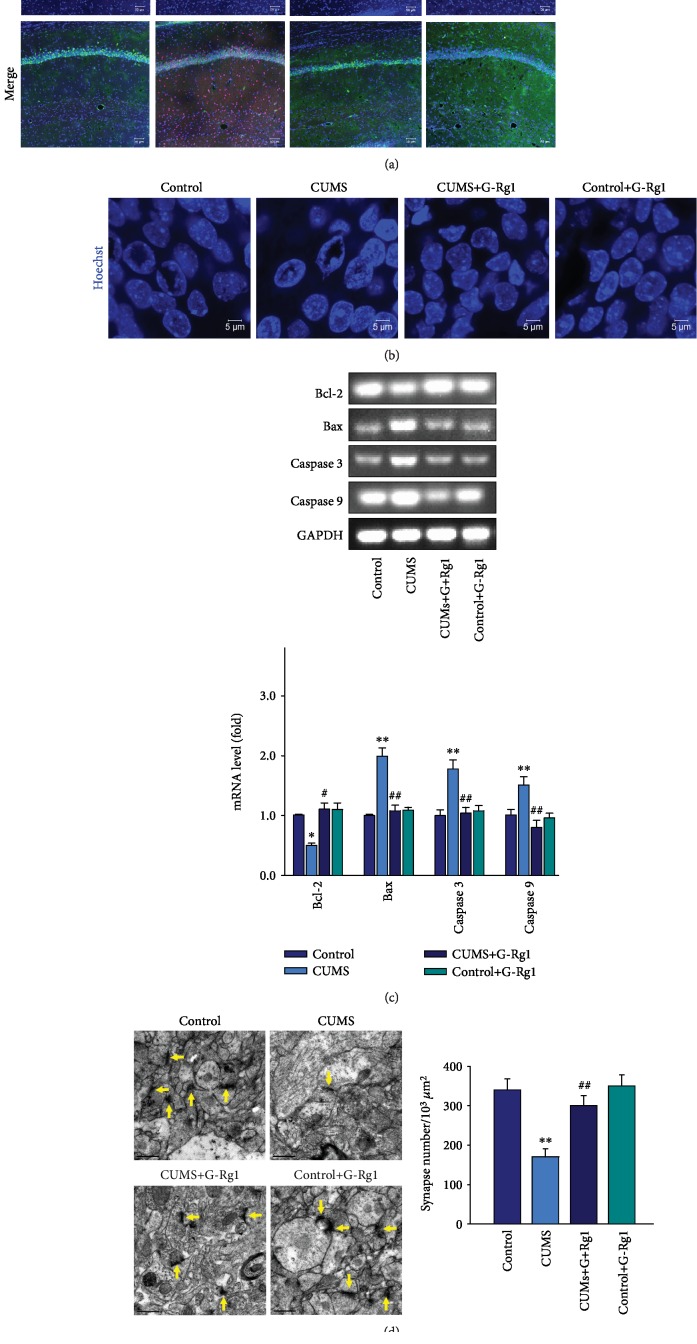
Ginsenoside-Rg1 decreases neural apoptosis in hippocampal CA1 regions of CUMS rats. (a) Ginsenoside-Rg1 pretreatment alleviated the increased double-labeled cleaved caspase 3/NeuN positive cells within the CA1 region of CUMS rats. Scale bar is 50 *μ*m. (b) Representative images of the Hoechst-33258 staining to observe morphological changes in nuclei. Ginsenoside-Rg1 pretreatment ameliorated the apoptotic nuclei within the CA1 region of CUMS rats. Scale bar is 5 *μ*m. (c) Ginsenoside-Rg1 pretreatment alleviated the mRNA expression levels of Bax, cleaved caspase 3, and caspase 9 within the CA1 region of CUMS rats. Band intensities were normalized to GAPDH. (d) Representative electron micrograph of CA1 neurons in rats. Arrows indicate spine synapses. Scale bar is 0.2 *μ*m. (e) Golgi staining images of the spine densities of dendrites within CA1 regions. Scale bar is 10 *μ*m. *N* = 6 per group. ^∗^*P* < 0.05 and ^∗∗^*P* < 0.01, compared to the control group; ^#^*P* < 0.05 and ^##^*P* < 0.01, compared to the CUMS group. G-Rg1: ginsenoside-Rg1.

Increased activation of oxidative stress and/or inflammation may result in cell death or senescence. Therefore, we next examined the effects of ginsenoside-Rg1/control treatments upon apoptosis within the CA1 regions of these CUMS rats. An overall statistically significant difference was found among these four groups with regard to apoptosis (*F*_(3, 20)_ = 15.32, *P* < 0.01). The post hoc analysis indicated that the density of positive cleaved caspase 3 and NeuN double-labeled cells was significantly increased within the CA1 region of CUMS-exposed rats as compared with that of the nonstressed control group (*P* < 0.01; Figure 6(a)). Moreover, the Hoechst-33258 staining showed that the CUMS exposure groups exhibited nuclear chromatin margination, aggregation, and condensation, changes which are typical of apoptotic nuclei as compared with that of the nonstressed control group (Figure 6(b)). In addition, mRNA levels of the apoptosis-related proteins Bax, caspase 3, and caspase 9 were all significantly increased within the CA1 region of depressed rats as compared with that of the nonstressed control group (*P* < 0.01 for all; Figure 6(c)). In contrast, these morphological changes associated with apoptosis and overexpression of proapoptotic factors within the CA1 region as induced by CUMS exposure were significantly suppressed in response to ginsenoside-Rg1 pretreatment (*P* < 0.01). Finally, the electron microscopy analysis showed that the ginsenoside-Rg1 pretreatment significantly ameliorated the synapse reduction in CA1 neurons caused by the CUMS exposure (*P* < 0.01; Figure 6(d)), while results from the Golgi staining revealed that dendritic spine loss in CA1 neurons resulting from CUMS exposure was significantly ameliorated by the ginsenoside-Rg1 pretreatment (*P* < 0.01; Figure 6(e)). There was no significant difference between the ginsenoside-Rg1-treated nonstressed control group and the nonstressed control group (*P* > 0.05). These results indicate that the neuroprotective effects of ginsenoside-Rg1 against apoptosis may contribute to the rescue of depression-like behaviors in CUMS rats.

## 4. Discussion

In the present study, we assessed the antidepressant-like effect of ginsenoside-Rg1 and explored its underlying mechanisms in a CUMS-induced rat model of depression. The results showed that ginsenoside-Rg1 treatment effectively prevented the development of depressive-like behaviors in CUMS rats. These beneficial effects on the behavior of these CUMS rats were accompanied with suppression of oxidative stress, as well as an attenuation in neuronal inflammation and apoptosis in the hippocampal CA1 area. Taken together, our data suggest that the neuroprotective effect of ginsenoside-Rg1 might serve as a potential therapy in the treatment of depression disorders.

Due to the similarities with that observed in major depression patients, the CUMS model has been widely used to induce depression-like behavior in animals as well as to evaluate the efficacy of chronic antidepressant treatments [26–28]. In the present study, we found that CUMS exposure decreased sucrose preference and increased immobility times in behavioral tests, results which were consistent with these previous studies. Of greater significance were the findings that ginsenoside-Rg1 pretreatment significantly prevented the depression-like behaviors of CUMS-exposed rats, similar to that observed in response to the classic antidepressant fluoxetine, and the identification of some of the mechanisms involved with these effects.

Results from previous animal as well as clinical studies have indicated that the limbic brain regions, including the prefrontal cortex, hippocampus, and amygdala, play a role in mood disorders [29, 30]. In the present study, we focused on the hippocampal CA1 region and found that CUMS exposure induces a lower degree of antioxidant enzyme activity while elevating oxidative stress levels at this brain site. In response to excessive ROS production, mitochondrial oxidative stress is enhanced as revealed by elevated levels of MitoSOX. And, these effects were accompanied by oxidative DNA damage as indicated by the DNA base damage marker, 8-OHdG. However, chronic pretreatment with ginsenoside-Rg1 inhibited the upregulation of oxidative stress and attenuated DNA damage induced by CUMS. In addition, we found that the NADPH oxidases, NOX1 and NOX4, which were upregulated by CUMS exposure, were downregulated by ginsenoside-Rg1 treatment. Such findings suggest that the antidepressant effects of ginsenoside-Rg1 may, in part, be attributable to decreased oxidative stress and possibly involve mediation via the NOX1/NOX4 pathway. With increased oxidative and nitrosative stress, there is an accumulation of toxic molecules including oxidizing lipids, proteins, and damaging nucleic acids which further activate neuronal inflammatory responses [31]. Results from a number of studies using animal models have indicated that increased inflammatory responses within the brain play a crucial role in the pathogenesis of depression [27, 32–34]. Similarly, findings from clinical studies have also reported the occurrence of atrophy and reduced hippocampal volume within the brain of patients with major depression disorders [35, 36]. Here, we show that CUMS exposure induces neuronal inflammation and apoptosis within the hippocampal CA1 region in rats, as well as producing a dramatic effect upon dendritic spine loss within the CA1 neurons. However, chronic treatment with ginsenoside-Rg1 in CUMS rats offered several beneficial effects including suppression of glia activation, downregulation of inflammatory cytokines, and attenuation of neuronal apoptosis. Such neuroprotective effects of ginsenoside-Rg1 may then contribute to these antidepressant-like effects. These observations suggest that a reciprocal relationship may exist between oxidative stress and neuroinflammatory responses which may then, in part, synergistically contribute to the neuronal jury and behavioral phenotypes underlying depression.

Taken together, we propose that the overactivation of oxidative stress may serve as a critical component in the pathogenesis of neurological diseases. Such a process has the capacity to produce damaged or dysfunctional proteins and organelles in neuronal cells, and thus promote neuroinflammation and apoptosis, eventually resulting in neural injury. Therefore, suppression of this neural process may represent one of the main mechanistic bases for the antidepressant-like effect of ginsenoside-Rg1. The identification of this mechanism may then serve as the foundation to identify more specific gene and protein expression alterations involved with the molecular mechanisms of ginsenoside-Rg1's ability to ameliorate CUMS-induced depressive behaviors.

## 5. Conclusion

In conclusion, this study provides strong evidence supporting the proposal that the rescue of CUMS-induced depressive-like behaviors by ginsenoside-Rg1 may be mediated by its neuroprotective effects resulting in anti-inflammatory and antiapoptosis effects within the hippocampal CA1 region. Effects which appear to, at least in part, be performed through suppression of the oxidative stress pathway. These findings revealed some of the underlying mechanisms for ginsenoside-Rg1's neuroprotective effects against neuronal injury and provide further insights into the potential for new therapeutic treatments of depression.

## Data Availability

The data used to support the findings of this study are available from the corresponding authors upon request.

## Abbreviations

ANOVA: Analysis of variance
CUMS: Chronic unpredictable mild stress
DMSO: Dimethyl sulfoxide
FST: Forced swim test
GFAP: Glial fibrillary acidic protein
ELISA: Enzyme-linked immunosorbent assay
IFN-*γ*: Interferon-*γ*
IL-1: Interleukin 1
i.p.: Intraperitoneal
LPS: Lipopolysaccharide
MDD: Major depressive disorder
RT-PCR: Reverse transcription PCR
SPT: Sucrose preference test
TNF-*α*: Tumor necrosis factor-*α*
4-HNE: 4-Hydroxynonenal.

## Supplementary Materials

Supplementary Materials Supplementary Table 1: PCR primers used in this study. Supplementary Figure 1: depression animal model experimental design: schematic figure of the treatment protocol of rats.
