# Supplementary material for: Ginsenoside-Rg1 Rescues Stress-Induced Depression-Like Behaviors via Suppression of Oxidative Stress and Neural Inflammation in Rats
Source: Oxid Med Cell Longev. 2020 Mar 18;2020:2325391. doi: 10.1155/2020/2325391 (PMC7125461; doi:10.1155/2020/2325391)
Supplement: Supplementary Materials — Supplementary Table 1: PCR primers used in this study. Supplementary Figure 1: depression animal model experimental design: schematic figure of the treatment protocol of rats. [file 2325391.f1.pdf]

.

Supplemental Table 1. PCR primers used in this study

Gene	*Forword (5'→3')*	*Reverse (5'→3')*
IL-1β	AAG ATG AAG GGC TGC TTC CAA ACC	ATA CTG CCT GCC TGA AGC TCT TGT
IFN-γ	ATT CAT GAG CAT CGC CAA GTT C	TGA CAG CTG GTG AAT CAC TCT GAT
TNF-α	TGA TCG GTC CCA ACA AGG A	TGC TTG GTG GTT TGC TAC GA
Bcl-2	GGA TCC AGG ATA ACG GAG GC	ATG CAC CCA GAG TGA TGC AG
Bax	TCT TCA AAC TGC TGG GCC ATT	CTT GTC ACC TGC CTG ACT GCT
Caspase3	GGA GCT TGG AAC GCG AAG AA	ACA CAA GCC CAT TTC AGG GT
Caspase9	CAA GAA GAG CGG TTC CTG GT	CAG AAA CAG CAT TGG CGA CC
NOX1	CCT GAA GGA TCC CAT CAG AGA	TGG AGG TCT GGA GCC TCT TA
NOX4	CCG GAC AGT CCT GGC TTA TC	TTG AGG GCA TTC ACC AAG TG
GAPDH	AGT GCC AGC CTC GTC TCA TA	GGT AAC CAG GCG TCC GAT AC
